# The thrombin receptor PAR4 supports visceral adipose tissue inflammation

**DOI:** 10.1007/s00210-024-03097-5

**Published:** 2024-04-23

**Authors:** Sonja Kleeschulte, Vivien Fischinger, Lisa Öhlke, Johannes Bode, Markus Kamler, Dobromir Dobrev, Maria Grandoch, Anke C. Fender

**Affiliations:** 1 Department of Gastroenterology, Hepatology and Infectious Diseases, University Hospital Düsseldorf, Düsseldorf, Germany; 2 Institute for Pharmacology and Clinical Pharmacology, University Hospital Düsseldorf, Düsseldorf, Germany; 3 Institute for Pharmacology, West German Heart and Vascular Center, University Duisburg-Essen, Duisburg, Germany; 4 Department of Medicine and Research Center, Montreal Heart Institute and Université de Montréal, Montréal, Canada; 5 Department of Integrative Physiology, Baylor College of Medicine, Houston, TX USA; 6 Department of Thoracic and Cardiovascular Surgery, University Hospital Essen, Essen, Germany; 7 Institute for Translational Pharmacology and CARID Cardiovascular Research Institute Düsseldorf, Medical Faculty and University Hospital Düsseldorf, Heinrich Heine University Düsseldorf, Düsseldorf, Germany

**Keywords:** Obesity, Adipose tissue, Inflammation, Glucose intolerance, Protease-activated receptor, Thrombin

## Abstract

Thrombin inhibition suppresses adiposity, WAT inflammation and metabolic dysfunction in mice. Protease-activated receptor (PAR)1 does not account for thrombin-driven obesity, so we explored the culprit role of PAR4 in this context. Male WT and PAR-4^-/-^ mice received a high fat diet (HFD) for 8 weeks, WT controls received standard chow. Body fat was quantified by NMR. Epididymal WAT was assessed by histology, immunohistochemistry, qPCR and lipase activity assay. 3T3-L1 preadipocytes were differentiated ± thrombin, acutely stimulated ± PAR4 activating peptide (AP) and assessed by immunoblot, qPCR and U937 monocyte adhesion. Epicardial adipose tissue (EAT) from obese and lean patients was assessed by immunoblot. PAR4 was upregulated in mouse WAT under HFD. PAR4^-/-^ mice developed less visceral adiposity and glucose intolerance under HFD, featuring smaller adipocytes, fewer macrophages and lower expression of adipogenic (leptin, PPARγ) and pro-inflammatory genes (CCL2, IL-1β) in WAT. HFD-modified activity and expression of lipases or perilipin were unaffected by PAR4 deletion. 3T3-L1 adipocytes differentiated with thrombin retained Ki67 expression, further upregulated IL-1β and CCL2 and were more adhesive for monocytes. In mature adipocytes, PAR4-AP increased phosphorylated ERK1/2 and AKT, upregulated Ki67, CCl2, IL-β and hyaluronan synthase 1 but not TNF-α mRNA, and augmented hyaluronidase-sensitive monocyte adhesion. Obese human EAT expressed more PAR4, CD68 and CD54 than lean EAT. PAR4 upregulated in obesity supports adipocyte hypertrophy, WAT expansion and thrombo-inflammation. The emerging PAR4 antagonists provide a therapeutic perspective in this context beyond their canonical antiplatelet action.

## Introduction

Obesity with central adiposity and ectopic fat deposition in visceral organs including the heart is a critical risk factor for development of type 2 diabetes, atherosclerosis and cardiovascular diseases (Neeland et al. 2019, Konwerski et al. 2022, Fang et al. 2023, Gawałko et al. 2023). Obesity is often accompanied by low-grade inflammation together with a hypercoagulant state. Thrombin activation increases in obese humans (Beijers et al. 2012, Prüller et al. 2012, Chitongo et al. 2017) and in experimental models of visceral obesity such as the high fat diet (HFD)-fed mouse (Kaji et al. 2013, Kopec et al. 2014, Miszta et al. 2020). Even children with adiposity, who have not yet developed the chronic diseases typically associated with adult-onset metabolic impairment, exhibit activated coagulation and elevated levels of systemic pro-inflammatory cytokines (Stoppa-Vaucher et al. 2012). Increased procoagulant activity has also been demonstrated within adipose tissue from obese and type 2 diabetic patients and at the adipocyte level (Edén et al. 2019). Adipocytes express tissue factor (TF), prothrombin and coagulation factor FVII, and can themselves support coagulation factor Xa-triggered prothrombin activation (Edén et al. 2019, Blencowe et al. 2021). F2, the gene encoding prothrombin, was recently identified as a novel key regulatory hub for adipocyte lipid regulation during obesity development (Blencowe et al. 2021). Accordingly, mice with mutated TF are protected from HFD-induced obesity and insulin resistance (Badeanlou et al. 2011), while mice with a pro-coagulant mutation of the thrombomodulin gene that increases thrombin activity develop accelerated obesity under HFD, associated with localized deposition of pro-inflammatory fibrin in WAT (Kopec et al. 2017). Direct thrombin inhibition with argatroban or dabigatran limits obesity development and its metabolic sequelae in mice in some (Mihara et al. 2010, Kopec et al. 2017), but not all studies (Feldmann et al. 2019). In the latter study (Feldmann et al. 2019), dabigatran however suppressed accumulation of pro-inflammatory macrophages in visceral white adipose tissue (WAT) and the secretion of inflammatory adipocytokines to the circulation, suggesting that thrombin inhibition may promote metabolically healthy WAT expansion.

Besides its canonical role in fibrin formation and haemostasis, thrombin elicits diverse cellular effects via protease-activated receptors (PAR), a family of four G-protein coupled receptors activated through proteolytic cleavage by coagulant, tryptic and inflammatory proteases. PAR are widely distributed and are critical mediators of thrombo-inflammation and tissue remodelling (Fender et al. 2017, Fender et al. 2019). PAR were first described on human platelets, where PAR1 mediates the initial activation required for haemostasis and PAR4 mediates the delayed and sustained activation in response to high thrombin levels (Coughlin 1999, French and Hamilton 2016). In mouse platelets, PAR4 is the major thrombin receptor, with PAR3 acting as a co-factor (Coughlin 1999). PAR2, although not a classic thrombin receptor, also responds to very high thrombin concentrations (Mihara et al. 2016). In human adipose tissue, PAR1 expression is restricted to the vascular stroma and mature adipocytes, while PAR4 is predominant in preadipocytes and adipocytes (Strande and Phillips 2009). Thrombin stimulates the secretion of the CC-chemokine ligand 2 (CCL2, or monocyte chemotractant protein MCP-1) and vascular endothelial growth factor (VEGF) from preadipocytes, and the secretion of IL-1β, IL-6 and tumor necrosis factor (TNF)-α from adipocytes (Strande and Phillips 2009). Although PAR1 is considered as the predominant PAR that mediates the release of inflammatory cytokines like IL-6 from adipocytes (Kajimoto et al. 2012), it does not appear to contribute to obesity development under HFD in vivo (Badeanlou et al. 2011, Kopec et al. 2017), pointing to a contribution of other PAR subtypes to the thrombin actions in obesity. Here we tested the hypothesis PAR4 mediates the stimulatory effects of thrombin on visceral adiposity and inflammation.

## Methods

### Materials

Unless otherwise stated, materials were purchased from Merck KGaA/Sigma-Aldrich (Taufkirchen, Germany). Human α-thrombin was obtained from American Diagnostica GmbH (Pfungstadt, Germany), PAR4 activating peptide (PAR4-AP, AYP-NH_2_) from MedChemTronica (Sollentuna, Sweden), hyaluoronidase (streptomyces hyalurolyticus) from Sigma-Aldrich and calcein-AM from Calbiochem (Darmstadt, Germany).

### Mouse model of diet-induced adiposity

PAR-4^-/-^ (C57Bl/6J background) mice were a generous gift from Dr. Justin Hamilton (Australian Centre for Blood Diseases, Monash University, Australia) and bred in-house as a heterozygous line. Mice received water ad libitum and were kept at a 12 h light/dark cycle. From age 6 weeks, male wildtype (WT) were randomized to receive standard chow or a high fat diet (HFD, #S7200-E010), both from ssniff Spezialdiäten GmbH (Soest, Germany) for 8 weeks; HFD was also fed to PAR-4^-/-^ littermates. This model is conventionally seen to replicate the metabolic features of clinical obesity and type 2 diabetes (Bastías-Pérez et al. 2020, Aravani et al. 2021, de Moura et al. 2021). Male mice were chosen for this first-time exploration of PAR4 involvement in obesity since female mice frequently show resistance to diet-induced weight gain and metabolic derailment (Pettersson et al. 2012, Casimiro et al. 2021, Maric et al. 2022) which may skew outcome of PAR4 deletion. All animal experiments were performed in accordance with the ARRIVE and IMPROVE guidelines and the animal welfare guidelines of the University Duisburg-Essen. The experimental procedures were approved by the local state authority State Agency for Nature, Environment and Consumer Protection (Landesamt für Natur, Umwelt und Vebraucherschutz, LANUV) in North Rhine-Westphalia (Az. 84.02.04.2012/A366).

### Assessment of metabolic parameters and tissue collection

Animals were weighed weekly. Fat mass (% of fat plus lean mass) was determined during weeks 2, 4 and 8 of feeding using the Minispec NMR analyser (Bruker Corporation, Billerica, MA, USA) as described (Feldmann et al. 2019). Food intake was monitored daily; average food consumption was estimated by weighing the provided and remaining food and dividing the difference by the number of mice per cage. After 8 weeks, animals were fasted for 6 h and tail-vein blood glucose determined using the *Accu-Chek Compact Plus* Glucometer (Roche Diagnostics, Mannheim, Germany). Mice were sacrificed with CO_2_ and body length measured from nose to anus. Visceral (epididymal) white adipose tissue (WAT) was removed, weighed and either snap-frozen in liquid nitrogen and stored at -80°C or placed in formalin for histological analysis.

### Oral glucose tolerance testing (GTT)

GTT was performed during week 6 of feeding as described (Feldmann et al. 2019) by an observer blinded to treatment. Mice were fasted for 6 h prior to measurement of fasting tail-vein blood glucose as above. For oral GTT, mice were administered 1 g/kg glucose solution per os and blood glucose concentration measured after 5, 15, 30, 60 and 120 min. For quantification, the area under the glucose-time curve (AUC) was calculated.

### Histology and immunohistochemistry

Tissues were dehydrated in formalin for 24 h before embedding in paraffin overnight. Sections were prepared from a total of 3 depths, all 300 µm apart, to ensure assessment of the complete fat pad. Sections were mounted and dried at room temperature for 24 h, heat fixed at 60°C for 60 min then stained with haematoxylin & eosin (H&E). Macrophages were stained with anti-mouse galectin-3 (Mac2) antibody (Cedarlane, Burlington, Ontario, Canada), and visualized by brown DAPI staining upon addition of the HRP-coupled secondary antibody (Novus Biologicals, Littleton, CO, USA). Adipocyte size and area were assessed by an observer blinded to treatment with automated counting using ImageJ and a Fiji application developed in-house. For this, two randomly selected sections from each of the three regions within each tissue were scanned with the Axioimager.M2 microscope (Carl Zeiss Microscopy GmbH, Jena, Germany) and loaded into the program at 10X magnification. Non-adipocyte structures were deleted, and size parameters of complete adipocytes only were analysed. Mean values were obtained from the total of six sections per organ. Adipocyte sizes were subdivided into size classes to determine the frequency of distribution of the adipocyte size range. Accumulated macrophages around adipocytes in crown-like structures (CLS) (Altintas et al. 2011) were counted and mean counts normalized to CLS per 100 adipocytes.

### Lipase activity

Lipase activity in fresh adipose tissue was measured with a commercial kit (Biovision, Milpitas, CA, USA) as instructed by the manufacturer.

### 3T3-L1 adipogenic cell line

3T3-L1 preadipocytes (European Collection of Cell Cultures) were cultured in DMEM containing 4,5 g/l D-glucose, 10 % FBS and 1% penicillin/streptomycin (P/S, all from ThermoFischer Scientific, Darmstadt, Germany). Cells were passaged at maximal 70% confluence to avoid contact inhibition and used up to the 12^th^ passage, after which they failed to fully differentiate. Cells seeded for study were allowed to reach confluence, two days later differentiation was initiated with a conventional cocktail containing insulin, dexamethasone and IBMX (Roberts et al. 2009). After 48 h, medium was exchanged and supplemented with insulin only. Thereafter, cells were maintained in normal medium containing 10% FBS, replaced every 2 days. Thrombin or PBS were first added with differentiation cocktail and replaced together with fresh medium every 2 days. Cells were harvested for study on day 7 of differentiation.

### Preadipocyte proliferation

Undifferentiated 3T3-L1 preadipocytes were seeded into 24-well plates at 5000 cells/well ± thrombin (1 U/mL) or PAR4-AP (100 µM), with six replicates per condition and time-point. Cell numbers per well were counted every 24 h. Wells with visible cell detachment were discarded. After careful washing, cells were detached with 200 µL trypsin-EDTA (ThermoFischer Scientific) for exactly 2 min at 37°C and the reaction stopped with addition of 50 µL FBS. All wells with incomplete detachment were discarded. Cell number from remaining samples (generally ≥ 3 replicates per condition) was determined by trypan blue exclusion using the Countess® Automated Cell Counter (ThermoFischer Scientific).

### Monocyte adhesion assay

The human leukemic monocyte lymphoma cell line U937 (CLS Cell Lines Service GmbH, Eppelheim, Germany) was used for determination of monocyte adhesion to differentiated 3T3-L1 adipocytes. For fluorescent labeling, 1x10^7^ cells in 1 mL RPMI medium (containing Glutamax and 1% P/S, all from ThermoFisher) were loaded with 10 µg/ml calcein-AM for 30 min at 37°C in the dark, being inverted once after 15 min. Labelled U937 cells were washed three times with 1 ml RPMI and resuspended to 3x10^6^ cells /mL in RPMI. Aliquots of 100 µL containing 3x10^5^ U937 cells were added to each well containing 3T3-L1 previously differentiated ± thrombin or PAR4-AP. Some adipocytes were additionally pretreated with hyaluoronidase (2 U/mL, 1 h). Labelled monocytes were allowed to adhere to adipocytes for 90 min at 4 °C in the dark. Wells were washed three times with ice-cold PBS, then replenished with 100 µL RPMI and calcein fluorescence was analysed using the Infinite® 200 plate-reader (Tecan Group Ltd., Männedorf, Switzerland) at excitation of 485 nm and emission of 535 nm. Experiments were performed as triplicates.

### Ectopic fat from human atrial biopsies

Epicardial adipose tissue attached to right atrial appendages was obtained from obese (BMI ≥ 30) and lean (BMI ≤ 25) patients undergoing open-heart surgery for coronary bypass grafting or valve replacement. Each patient gave written informed consent. Tissue samples were collected immediately prior to atrial cannulation for extracorporeal circulatory bypass, stored in Tyrode solution and transferred to the laboratory for freezing. Individual clinical patient characteristics are depicted in Table 1. The studies were approved by the Human Ethics Committee of the Medical Faculty of the University Duisburg-Essen (approval number AZ:12-5268-BO) and were performed in accordance with the Declaration of Helsinki.

**Table 1:** Patient characteristics

	Lean	Obese
Patients, n	6	6
Male gender, n (%)	4 (67)	4 (67)
Age (years), mean±SD	69±6	70±7
BMI (kg/m^2^), mean±SD	24±2	33±2
CAD, n (%)	0 (0)	3 (50)
AVD/MVD, n (%)	3 (50)	0 (0)
CAD + AVD/MVD, n (%)	3 (50)	3 (50)
Hypertension, n (%)	3 (50)	2 (33)
Hyperlipidaemia, n (%)	1 (17)	1 (17)
Smoker – current or ex, n (%)	0 (0)	0 (0)
Previous MI, n (%)	0 (0)	0 (0)
LVEF (%), mean±SD	67±5	58±2
ACEI/ARB, n (%)	3 (50)	3 (50)
Beta blockers, n (%)	3 (50)	5 (83)
Calcium channel blockers, n (%)	1 (17)	2 (33)
Diuretics, n (%)	2 (33)	2 (33)
Lipid-lowering drugs, n (%)	4 (67)	4 (67)
Oral anticoagulants, n (%)	0 (0)	0 (0)
Acetylsalicylic acid, n (%)	4 (67)	6 (100)

*ACEI*, angiotensin converting enzyme inhibitors; *ARB*, angiotensin receptor blockers; *AVD*, aortic valve disease; *BMI*, body mass index; *CAD*, coronary artery disease; *LVEF*, left ventricular ejection fraction; *MI*, myocardial infarction; *MVD*, mitral valve disease; *SD*, standard deviation

### mRNA expression analysis

Total RNA was trizole-extracted (peqGOLD TriFast, Peqlab, Erlangen, Germany) from cells and frozen tissues as instructed by the manufacturer. Adipose tissue samples were shredded using an RNase-free tungsten carbide-grinding ball in an MM 400 oscillating mill (Retsch GmbH, Haan, Germany). Purity and concentration of RNA preparations were verified (Nanodrop 2000, ThermoFischer Scientific) prior to transcription into cDNA using the QuantiTect Reverse Transcription Kit (Qiagen, Hilden, Germany) according to the manufacturer’s instructions. Target mRNA expression was quantified by an observer blinded to treatment using Validated Quantitect Primer Assays (Qiagen) and the StepOnePlus Real-Time PCR System using Platinum SYBR Green qPCR SuperMix-UDG (ThermoFischer Scientific). Target mRNA levels were normalized to ribosomal 18S by the ΔΔCT method.

### Protein expression analysis

Cells were lysed in Kranias buffer containing 1.5 M Tris (pH 8.8), 0.5 M EDTA (pH 8.0), 1 M NaF, 20% SDS and 10% glycerol, and supplemented with 1:10 cOmplete™ Mini Protease Inhibitor Cocktail and 1:10 PhosSTOP™ Phosphatase Inhibitor Cocktail immediately prior to use. All chemicals were from Sigma-Aldrich. Frozen tissues were crushed under liquid nitrogen and homogenised in Kranias buffer and cleared by centrifugation (15 min, 900xg at room temperature). Protein content was assessed with the Pierce BCA Protein Assay Kit (ThermoFisher Scientific, Dreieich, Germany). 100 µL aliquots of cleared lysates were supplemented with 20 µL of 6x Laemmli buffer and heated to 95°C for 5 min. Western blotting was then performed by an observer blinded to treatment as described (Fender et al. 2020, Scott et al. 2021). Primary antibodies used were PAR4 (#ab188930), and CD68 (#ab201973) from Abcam (Cambridge, UK); CD54 (#MA5407), AKT1 (#PA1-22099) and γ-tubulin from ThermoFisher); phospho-(Ser473)-AKT (#SAB3701426) from Sigma; phospho-(Thr202/Tyr204)-ERK1/2 (#9101), ERK1/2 (#9102), phospho-(Thr172)-AMPKα (#2535) and AMPKα (#2532) from Cell Signaling Technology (Danvers, MA, USA). Infrared-coupled secondary antibodies were obtained from LI-COR Biosciences (Bad Homburg, Germany). All antibodies were diluted 1:1000. Band visualization and quantification was performed using the LI-COR Odyssey platform.

### Data and statistical analysis

Data are presented as mean ± SD, normalised to controls as indicated. Statistical analysis between two groups utilised Wilcoxon matched rank test. Comparison between three groups was performed using one-way analysis of variance (Kruskall-Wallis) with Dunn’s post hoc test for multiple comparisons, using GraphPad PRISM. *P*<0.05 was considered as statistically significant.

## Results

### PAR4 deletion retards visceral adiposity development under HFD

**Fig. 1:**
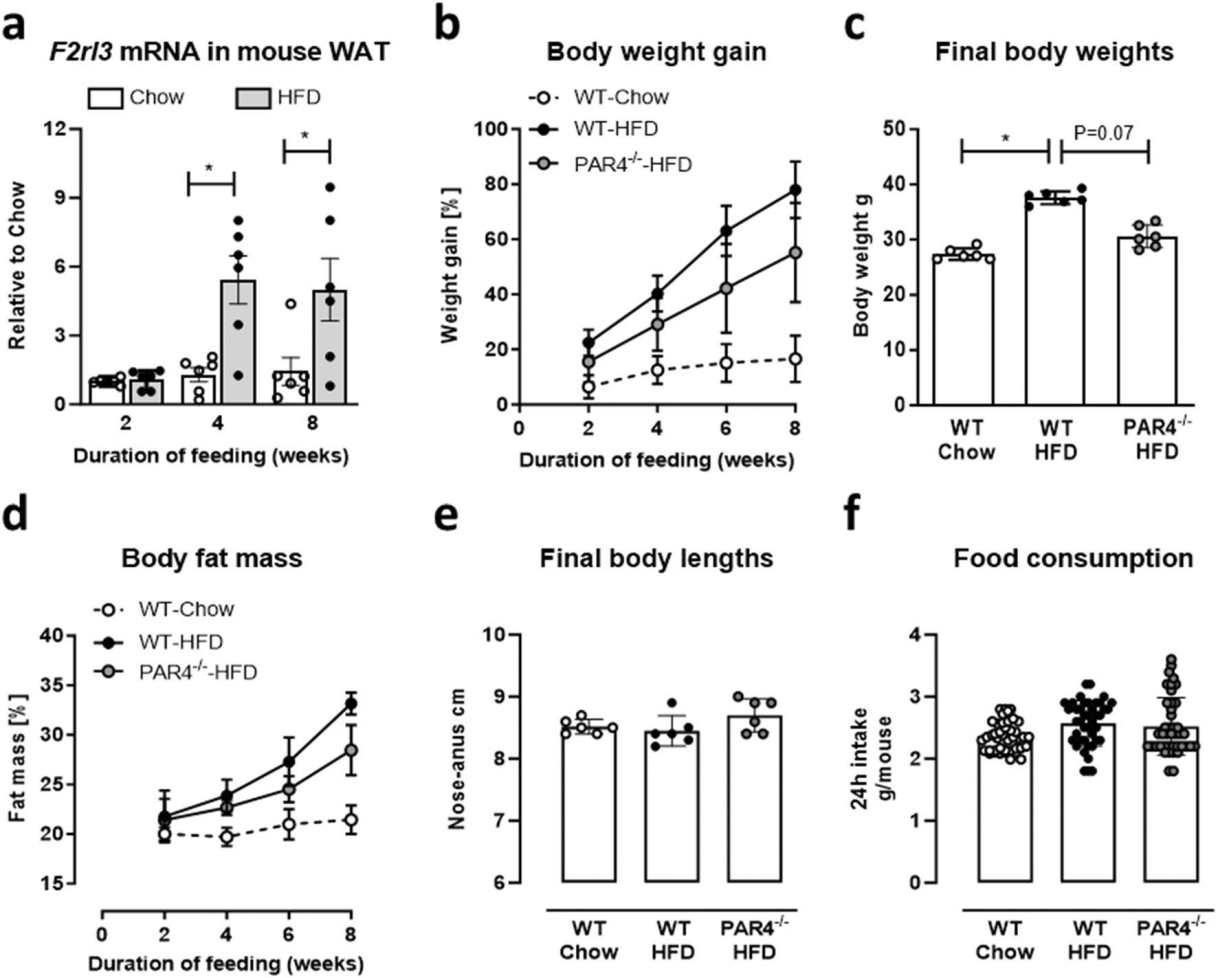
Mice lacking PAR4 are partically protected from visceral adiposity induced by high fat diet (HFD). **a** Transcript levels of the PAR4-encoding gene *F2rl3* in visceral epidydimal white adipose tissue (WAT) of mice fed HFD compared to chow. **b** Progressive body weight gain (as % of start weight) in wildtype (WT) mice fed chow or HFD and PAR4^-/-^ littermate mice fed HFD for up to 8 weeks. **c** Body weights at the start and end of the 8-week feeding intervention in the three groups of mice. **d** Progressive accumulation of body fat mass (as % lean plus fat mass) determined by NMR spectroscopy in the three groups. **e** Body lengths measured from nose to anus at the start and end of the 8-week feeding intervention in the three groups of mice. **f** Average 24 h food consumption per mouse over the 8-week intervention in the three groups. Data show mean ± SD, *denotes *P*<0.05

Wildtype (WT) mice fed a HFD for up to 8 weeks showed a progressive upregulation of PAR4 mRNA in visceral WAT compared to chow-fed littermates; the nearly 6-fold difference was significant from 4 weeks of feeding onwards (Fig. 1a, all *n*=6). Obesity development was assessed in three groups of mice: WT mice fed chow (start weight 22.4±2.5 g), WT mice fed HFD (start weight 22.25±1 g) and PAR4^-/-^ mice fed HFD (start weight 22.32±2.1 g) for up to 8 weeks. The HFD considerably accelerated weight gain in WT mice compared to mice maintained on standard chow (Fig. 1b), resulting in higher body weights at the end of the 8 weeks (Fig. 1c). Body fat mass also increased significantly in the HFD vs. chow group (Fig. 1d). Genetic deletion of PAR4 attenuated these measures of visceral obesity development under HFD. Body length at sacrifice (Fig. 1e) and average 24 h food consumption over the feeding period (Fig. 1f) did not differ between the groups.

### PAR4 deletion reduces adipocyte hypertrophy

**Fig. 2:**
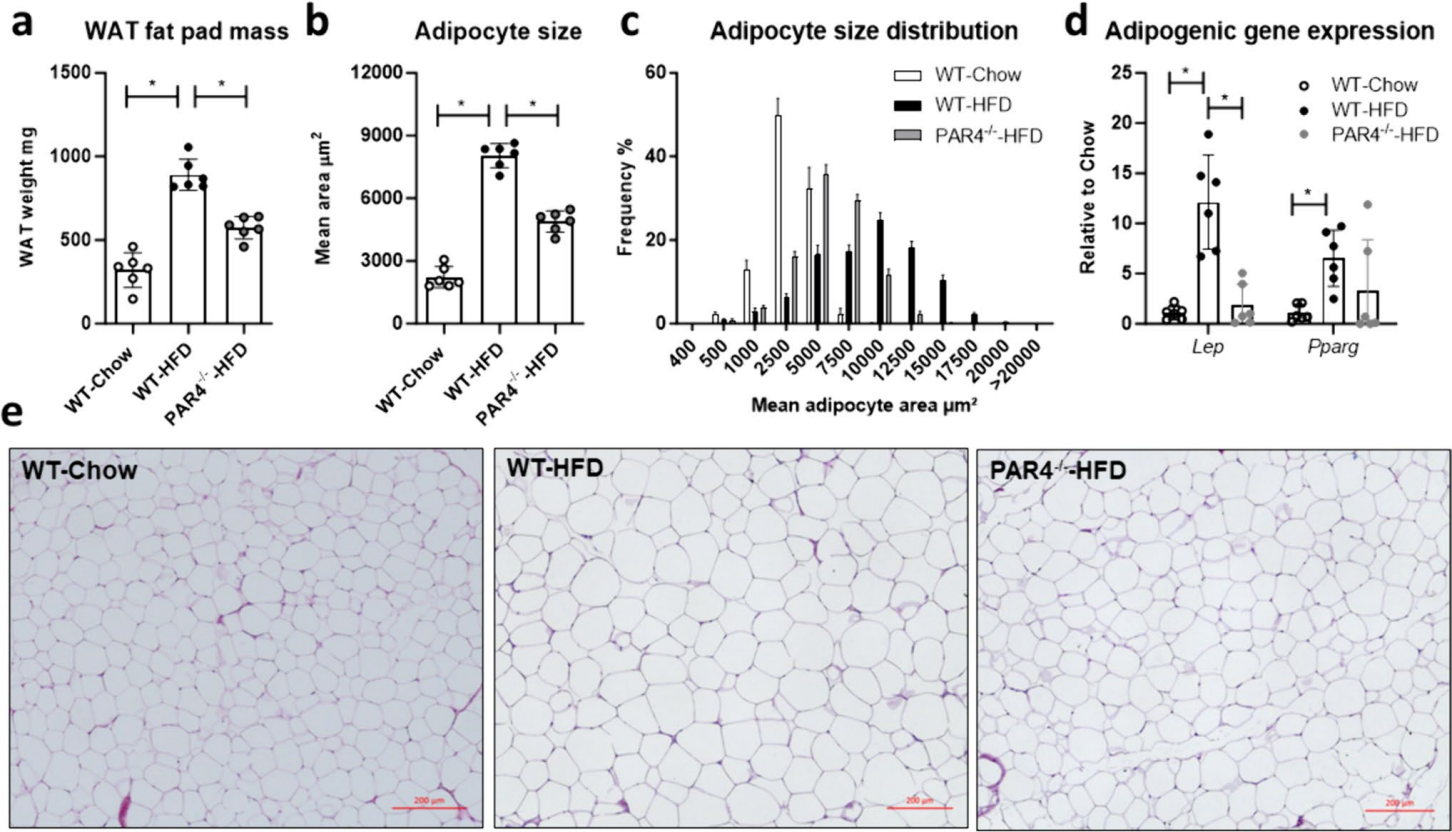
High fat diet (HFD)-induced adipocyte hypertrophy is blunted in PAR4^-/-^ mice. **a** Weight of the epidydimal white adipose tissue (WAT) fat pad in wildtype (WT) mice fed chow or HFD and PAR4^-/-^ littermate mice fed HFD for up to 8 weeks. **b** Average adipocyte area and **c** frequency distribution of mean adipocyte areas in the three groups at the end of 8 weeks. **d** Transcript expression of the adipogenic genes encoding leptin (*Lep*) and peroxisome proliferator-activated receptor gamma (*Pparg*) in visceral WAT in the three groups at the end of 8 weeks. **e** Representative histology of WAT from the three groups after 8 weeks. Data show mean ± SD, *denotes *P*<0.05

WAT mass, determined as weight of the epidydimal fat pad taken from the heart-side of the mouse at sacrifice, increased notably in WT mice fed a HFD, but less so PAR4^-/-^ mice (Fig. 2a). Mean adipocyte area was about 4-fold greater in WT mice fed HFD compared to chow, in HFD-fed PAR4^-/-^ mice 2-fold greater than in the controls (Fig. 2b). The size distribution frequency is depicted in Fig. 1c, representative histology in Fig. 2d. Gene expression of the adipogenic differentiation markers leptin and PPARγ increased in WAT of HFD-fed mice compared to chow-fed mice; this increase was not evident in PAR4^-/-^ mice (Fig. 2d).

### PAR4 deletion attenuates inflammation of visceral fat

**Fig. 3:**
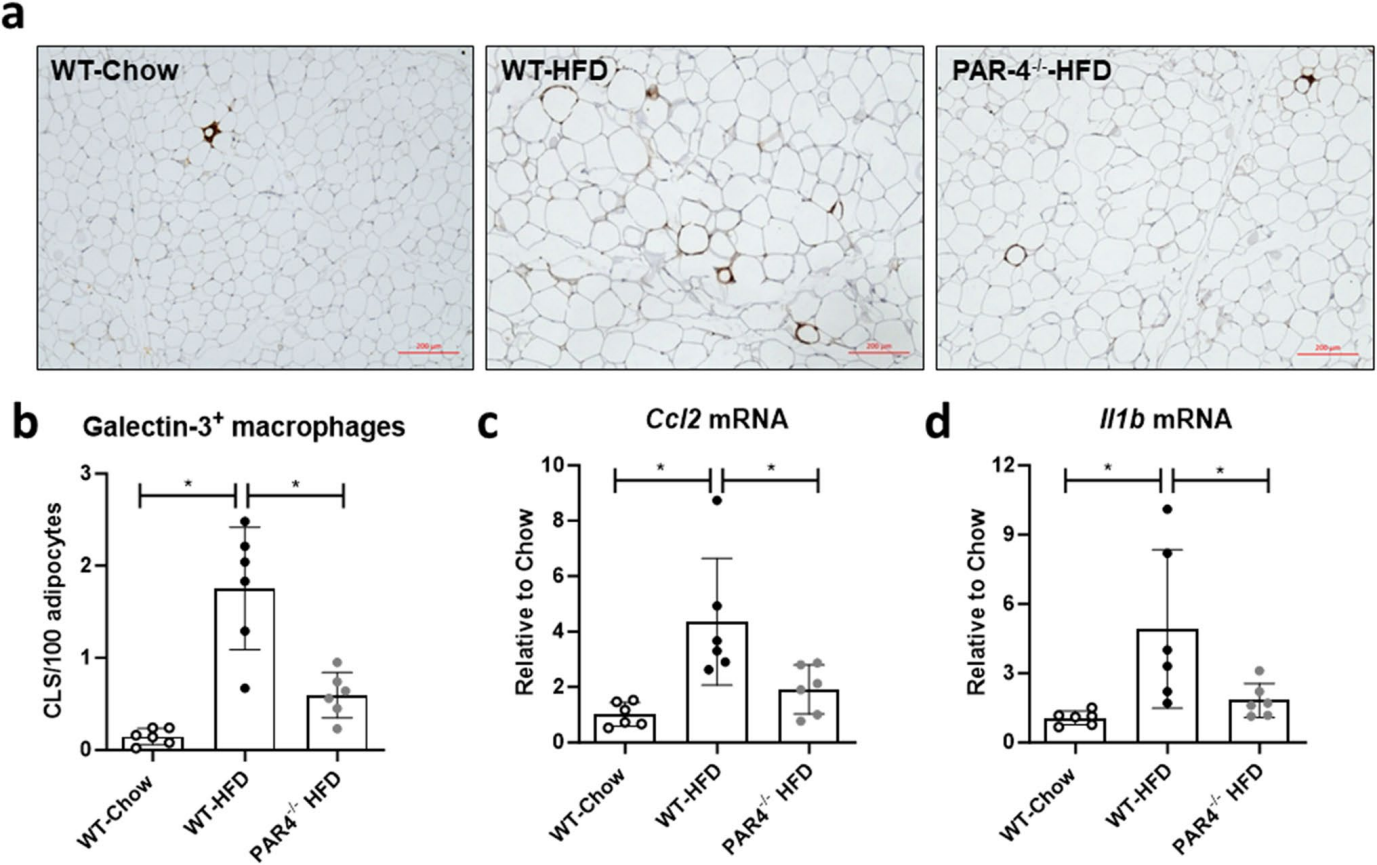
White adipose tissue (WAT) inflammation induced by high fat diet (HFD) is attenuated in PAR4^-/-^ mice. **a** Representative histology showing galectin-3 positive macrophages in epidydimal WAT of wildtype (WT) mice fed chow or HFD and PAR4^-/-^ littermate mice fed HFD for 8 weeks. **b** Quantification of galectin-3-stained macrophages clustered into crown-like structures (CLS), as number of CLS per 100 adipocytes, in WAT from the three groups after 8 weeks. **c** Transcript expression of the genes encoding CC-chemokine ligand 2 (*Ccl2*) and (d) IL-1β (*Il1b*) in WAT of the three groups of mice at 8 weeks. Data show mean ± SD, *denotes *P*<0.05

Galectin-3-stained macrophages, clustered into crown-like structures (CLS), were significantly more abundant in WAT from HFD-fed WT mice compared to controls and PAR4^-/-^ mice (Fig. 3a, b). Transcript levels of CCL2 and IL-1β (Fig. 3c, d) were about 4-fold higher in WAT from HFD-fed compared to controls and PAR4^-/-^ mice.

### PAR4 deletion improves glucose tolerance

**Fig. 4:**
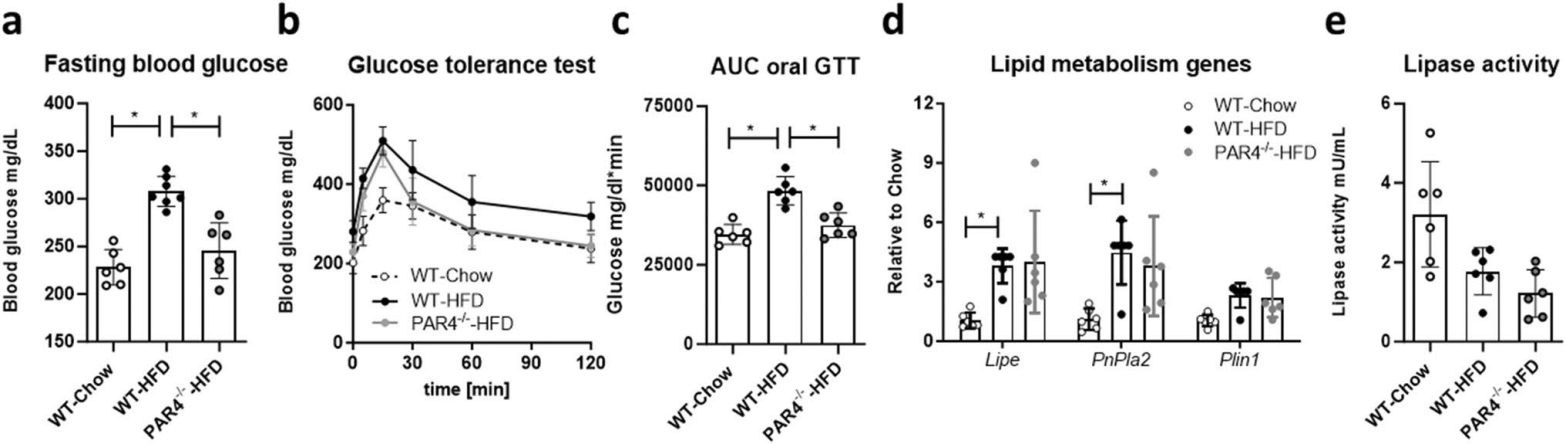
PAR4 deletion improves glucose tolerance in high fat diet-fed mice. **a** Fasting blood glucose measured in tail-vein blood of wildtype (WT) mice fed chow or HFD and PAR4^-/-^ littermate mice fed HFD for 6 weeks. **b** Blood glucose time-course after oral glucose challenge in the three groups of mice after 6 weeks of dietary intervention. **c** Area under the curve (AUC) of the oral glucose tolerance test (GTT) timecourse. **d** Transcript expression of the metabolic regulator genes encoding lipprotein lipase (*Lipe*), adipocyte triglyceride lipase (ATGL, *Pnpla2*) and perilipin (*Plin1*) in visceral WAT in the three groups after 8 weeks dietary intervention. **e** Lipase activity assay performed in fresh WAT from the three groups of mice after 8 weeks. Data show mean ± SD, *denotes *P*<0.05

Fasting blood glucose was higher in HFD-fed WT mice compared to WT mice on chow or PAR4^-/-^ mice on HFD (Fig. 4a). Oral glucose tolerance testing showed faster recovery of blood glucose in HFD-fed PAR4^-/-^ versus WT mice (Fig. 4b), the calculated AUC was comparably low in controls and PAR4^-/-^ mice and highest in HFD-fed WT mice (Fig. 4c). Lipoprotein lipase, perilipin and adipose triglyceride lipase (ATGL) mRNAs were comparably upregulated in WAT under HFD while lipase activity in fresh WAT was modestly attenuated, regardless of phenotype (Fig. 4d, e).

### PAR promote a pro-inflammatory proliferative adipocyte phenotype in vitro

**Fig. 5:**
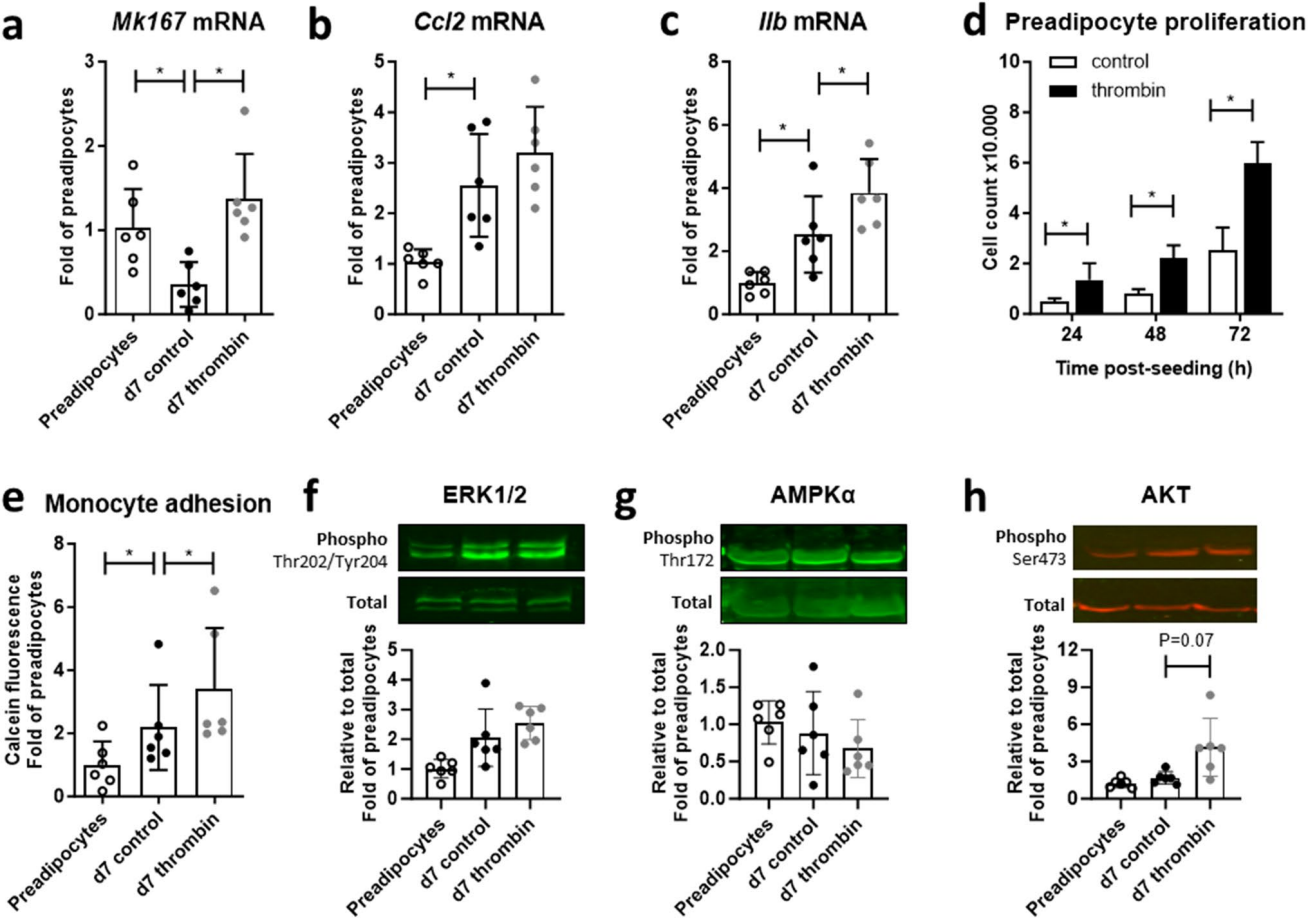
Proliferative and inflammatory adipocyte responses to thrombin. **a** Transcript levels of the genes encoding Ki67 (*Mk167*), **b** CC-chemokine ligand 2 (*Ccl2*) and **c** IL-1β (*Il1b*) in 3T3-L1 preadipocytes and adipocytes differentiated for 7 days ± thrombin. **d** Cell counts in preadipocytes seeded ± thrombin. **e** Adhesion of calcein-labelled U937 monocytes to preadipocytes and adipocytes differentiated ± thrombin. **f** Phosphorylated/total ERK1/2, **g** AMPKα and **h** AKT in preadipocytes and adipocytes differentiated for 7 days ± thrombin Data show mean ± SD, *denotes *P*<0.05

For mechanistic insight into thrombo-inflammation at the cellular level, the 3T3-L1 adipogenic cell line was treated ± thrombin throughout the differentiation time course. On day 7, cultures had downregulated expression of the proliferation marker Ki67 compared to preadipocytes, thrombin stimulation throughout differentiation retained Ki67 transcript expression (Fig. 5a). Expression of CCL2 and IL-1β mRNA was by contrast upregulated in differentiated versus undifferentiated adipocytes that was incrementally increased by thrombin, significantly so for IL-1β (Fig. 5b, c). The mitogenic action typical for thrombin was validated in preadipocytes seeded in the presence of thrombin, which accelerated cell proliferation (Fig. 5d). Mature adipocytes were more adhesive for U937 monocytes with greatest adhesion seen in thrombin-treated cultures (Fig. 5e). Mature adipocytes showed only modestly higher ERK1/2 phosphorylation at Thr202/Tyr204 than preadipocytes with no incremental increase with thrombin treatment (Fig. 5f). AMPKα phosphorylation at Thr172 was not notably different between the groups (Fig. 5g) but AKT Ser473-phosphorylation was detectably higher in adipocytes matured in the presence of thrombin (Fig. 5h).

**Fig. 6:**
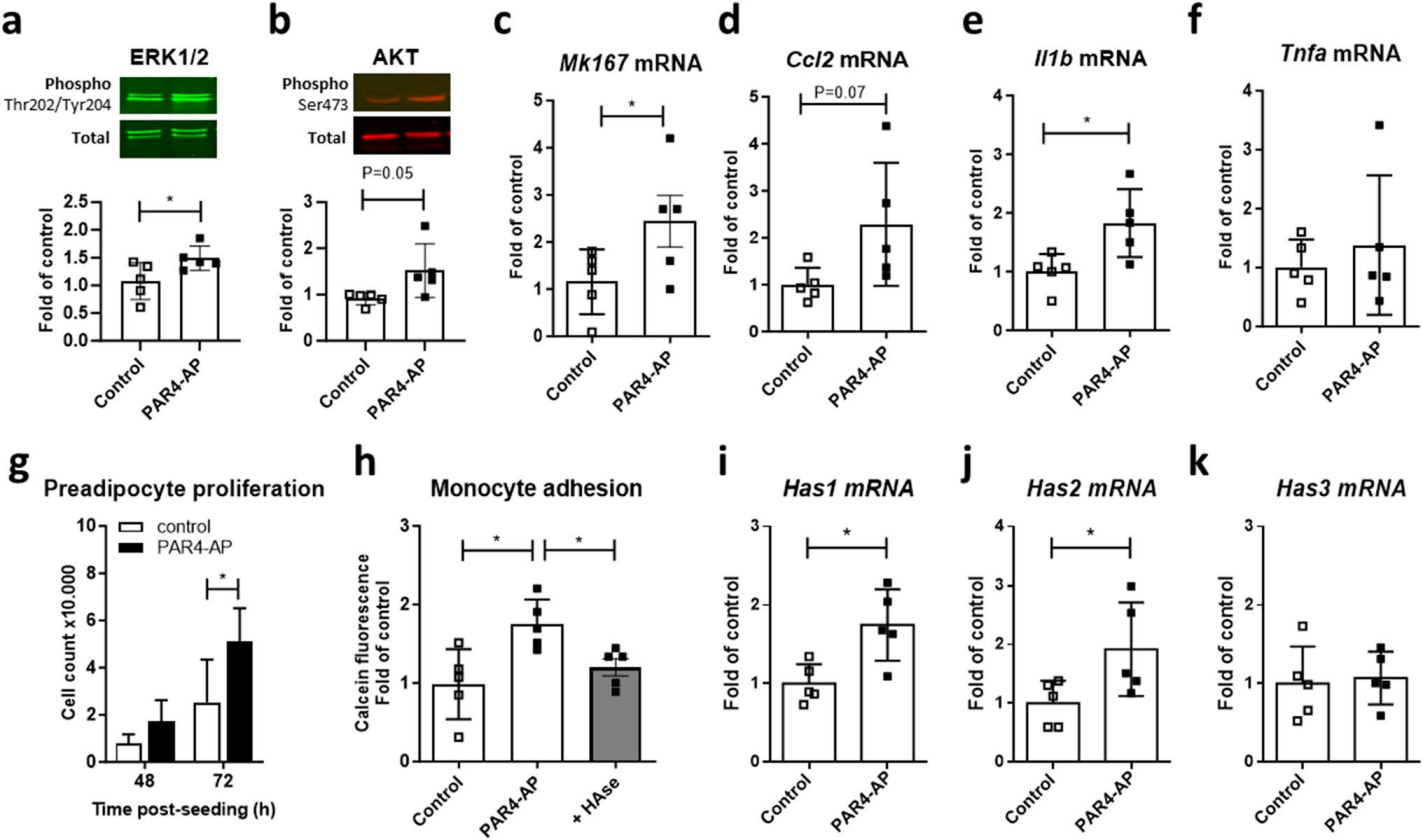
Proliferative and inflammatory adipocyte responses to PAR4 activation. **a** Phosphorylated/total ERK1/2 and **b** AKT in differentiated 3T3-L1 stimulated ± PAR4 activating peptide (AP, 100 µmol/L) for 30 min. **c** Transcript levels of the genes encoding Ki67 (*Mk167*), **d** CC-chemokine ligand 2 (*Ccl2*), **e** IL-1β (*Il1b*), **f** hyaluronan synthase 1 (*Has1*) and **g** tumor necrosis factor α (*Tnfa*) in differentiated 3T3-L1 stimulated ± PAR4-AP for 24 h. **h** Cell counts in preadipocytes seeded ± PAR4-AP. **i** Adhesion of calcein-labelled U937 monocytes to mature adipocytes pretreated ± PAR4-AP for 24 h. Data show mean ± SD, *denotes *P*<0.05

To specifically explore PAR4-mediated effects and avoid adaptive regulation during long-term thrombin exposure, mature adipocytes were acutely exposed to the PAR4 activating peptide (AP) or vehicle for different intervals. Selective PAR4 stimulation increased phosphorylation of ERK1/2 and AKT at 30 min (Fig. 6a, b) and upregulated of Ki67, CCl2, IL-β and hyaluronan synthase isoform HAS1 mRNAs over 24h (Fig. 6c-f). TNF-α mRNA was not regulated (Fig. 6g). Accelerated cell proliferation was confirmed in preadipocytes seeded in the presence of PAR4-AP (Fig. 6h). Monocyte adhesion to mature adipocytes was also greater after 24h pretreatment with PAR4-AP, and this effect was sensitive to hyaluronidase.

### PAR4 and macrophage markers increase in obese human ectopic fat

**Fig. 7:**
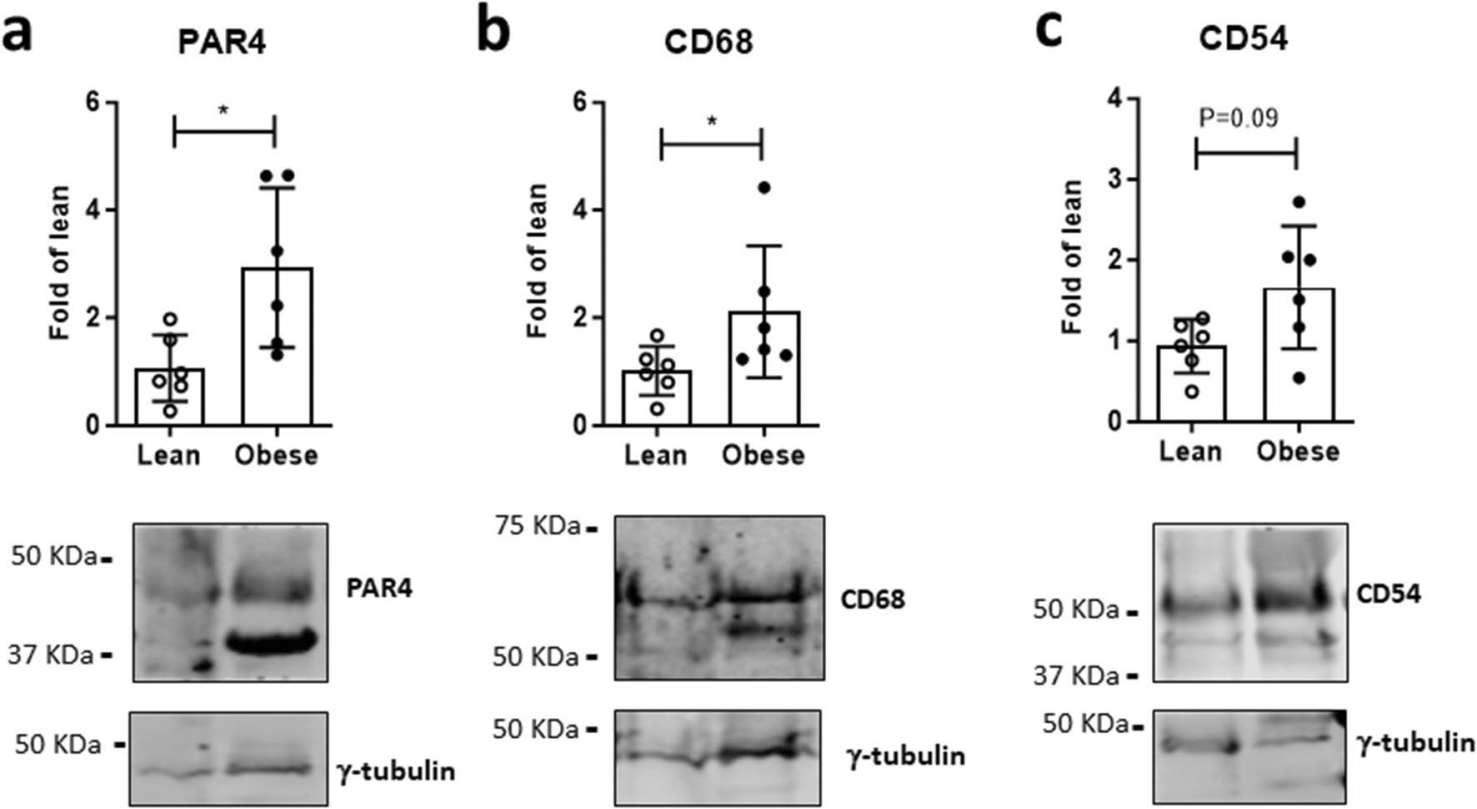
PAR4 coincides with macrophage markers in human epicardial fat. **a** Protein abundance of PAR4, **b** CD68 and **c** CD54 in epicardial adipose tissue from lean versus obese patients. Data show mean ± SD, *denotes *P*<0.05

To validate the results in the human, PAR4 and the macrophage markers CD68 and CD54 were determined in adipose tissue taken from human atrial appendages during open-heart surgery. Expression of all three proteins was higher in samples from obese compared to matched lean patients (Fig. 7).

## Discussion

Thrombin inhibition limits weight gain, adipose tissue inflammation and metabolic dysfunction in mouse models of obesity and diabetes (Mihara et al. 2010, Kopec et al. 2017). Mechanistically, this was attributed largely to suppression of pro-inflammatory fibrin deposition (Kopec et al. 2017). Thrombin signalling through its prototypical receptor PAR1 does not appear to contribute since PAR1^-/-^ mice showed no overt protection under HFD (Badeanlou et al. 2011, Kopec et al. 2017). Here we establish the causal involvement of PAR4, a low-affinity thrombin receptor, in adipose tissue inflammation and metabolic dysfunction (see graphical abstract). The so-called metaflammation might therefore constitute a potential indication for the novel PAR4 antagonists currently in clinical development (Merali et al. 2022, Merali et al. 2023).

PAR4, but not PAR1, is progressively upregulated in 3T3-L1 adipocytes undergoing differentiation (Feldmann et al. 2019). In the present study, PAR4 expression in WAT increased with obesity development in mice to least 5-fold over levels detected in chow-fed controls. The accelerated gains in weight and body fat in response to HFD were reduced in PAR4^-/-^ mice. Together, these observations strongly implicate PAR4 in early obesity. Neither body length at sacrifice nor the average food consumption over time differed between the groups, indicating that the changes seen were unlikely due to effects on growth or appetite. WT-mice on HFD exhibited greater WAT mass and larger adipocytes, but in PAR4^-/-^ mice, HFD-induced adipocyte hypertrophy and WAT expansion were markedly reduced, as was expression of the adipogenic genes for leptin and PPARγ.

The finding that PAR4 deletion resulted in smaller adipocytes is at odds with the effect of dabigatran, which increased WAT adipocyte size in *Ldlr*^-/-^ mice with HFD-induced obesity (Feldmann et al. 2019). In that study, dabigatran suppressed accumulation of pro-inflammatory M1 macrophages in WAT, thus promoting a metabolically healthy phenotype with low inflammation, despite an apparent adipocyte hypertrophy. In this study, PAR4^-/-^ mice exhibited lower macrophage accumulation in WAT under HFD, accompanied by near-complete suppression of the monocyte chemotractant CCL2. CCl2 critically supports macrophage recruitment to WAT, leading to local tissue inflammation and ultimately glucose intolerance and insulin resistance (Kamei et al. 2006). Accordingly, the representative pro-inflammatory cytokine IL-1β was also suppressed in WAT of PAR4^-/-^ mice under HFD, reflecting our recent report linking PAR4 with an overactive inflammasome in diabetic myocardium (Fender et al. 2020). The impaired glucose tolerance seen in HFD-fed WT mice was also improved by PAR4 deletion, in accordance with the metabolic protection afforded by thrombin inhibition in *db/db* or HFD-fed C57BL/J mice (Mihara et al. 2010, Kopec et al. 2017). Since thrombin has been reported to elicit a biphasic activation of lipoprotein lipase activity in rat primary adipocytes (Soma et al. 1989), we also examined lipase activity and genes related to lipid metabolism and compartmentalisation. Transcript levels of lipoprotein lipase, adipose triglyceride lipase and perilipin increased while lipase activity decreased in WAT of HFD-fed vs. control mice. Somewhat unexpectedly, PAR4 deletion had no effect on any of these parameters, indicating a selective preservation of glucose homeostasis with negligible impact on lipid metabolism.

Mechanistic insights into the cellular actions of thrombin/PAR4 were sought with the differentiating 3T3-L1 cell model of adipogenesis. 3T3-L1 preadipocytes stop proliferating and switch to an adipogenic gene programme upon reaching confluence. Maturation into lipid-filled adipocytes can be accelerated with a standard differentiation cocktail (Roberts et al. 2009). Mature adipocytes markedly downregulated Ki67 compared to preadipocytes, but expression was retained in cultures supplemented with thrombin every other day, indicating ongoing proliferative capacity. The mitogenic action of thrombin was validated in preadipocytes, which when seeded in thrombin-containing medium showed accelerated increases in cell numbers. CCl2 mRNA increased in differentiated vs. undifferentiated adipocytes, reflecting our finding in WAT from HFD-fed mice. Continued thrombin exposure raised CCL2 mRNA further, albeit non-significantly, and led to greater monocyte adhesion, consistent with the role of thrombin and its receptors in directing monocyte recruitment and macrophage accumulation (Mahajan-Thakur et al. 2014, Pavic et al. 2014, Feldmann et al. 2019). Thrombin-treated adipocytes also upregulated IL-1β mRNA, in accordance with our observations in WAT of HFD-fed mice and our previous findings in thrombin-stimulated human cardiac fibroblasts (Fender et al. 2020). Consistent with reports of increased ERK1/2 activity during adipocyte maturation in vitro and obesity development in vivo (Chen et al. 2022, Sun et al. 2023), we found a modestly elevated ERK1/2 phosphorylation in differentiated 3T3-L1. ERK1/2 is a classic effector of thrombin-stimulated mitogenesis, but the presence of thrombin during differentiation did not incrementally increase phosphorylated ERK1/2. This may be due to the acute nature of thrombin-induced ERK1/2 activation, which at least in vascular smooth muscle cells typically peaks at 5 min (PAR1-mediated) and again at 30–60 min (PAR4-mediated) (Pape et al. 2008, Dangwal et al. 2011, Pavic et al. 2014). Adipocyte differentiation and lipid deposition during diet-induced obesity have both been linked with an overactive AKT/mTOR pathway and suppression of AMPKα activity (Gurriarán-Rodríguez et al. 2011, Sun et al. 2023). In our matured adipocytes, AMPKα phosphorylation was not modified by either adipogenic differentiation per se or by thrombin. AKT however showed a modest increase in phosphorylation at least in thrombin-treated adipocytes, reflecting suppression of AKT signaling in argatroban-treated obese mice (Mihara et al. 2010).

Given our prior observation of progressively increased PAR4 expression in maturing 3T3-L1 (Feldmann et al. 2019), we examined the acute response of differentiated adipocytes to the selective PAR4 agonist peptide (AP) AYPGKV-NH_2_. In mature adipocytes, PAR4-AP increased phosphorylated ERK1/2 and AKT within 30 min and over 24 h elevated gene expression of Ki67, indicating a return to proliferative capacity. Accordingly, PAR4-AP was comparably mitogenic for preadipocytes as thrombin. Gene expression of CCL2 was also increased, and consistent with this, adipocytes pretreated with PAR4-AP attached monocytes more avidly than control adipocytes. While IL-1β mRNA was upregulated, as seen with thrombin, TNF-α mRNA was not, suggesting specific activation of the NLRP3 inflammasome. HAS1 transcript levels were also higher in adipocytes exposed to PAR4-AP. This enzyme is responsible for the production and extrusion of the extracellular matrix component hyaluronan, which contributes causally to adipose tissue inflammation and expansion (Grandoch et al. 2019, Misiou et al. 2021). In our study, hyaluronidase prevented the stimulatory effect of PAR4-AP on adipocyte/monocyte adhesion, in accordance with the creation of a monocyte-adhesive matrix formed by adipocyte-secreted hyaluronan (Han et al. 2007).

For a translational perspective, PAR4 and the macrophage markers CD68 and CD54 were assessed in atrial epicardial adipose tissue (EAT) from patients undergoing cardiac surgery. EAT is a specific type of visceral adipose tissue linked with diverse cardiovascular sequelae of obesity (Fang et al. 2023, Gawałko et al. 2023). In healthy conditions, EAT has a primarily protective function against mechanical stress or hypothermia, and supplies the myocardium with energy from free fatty acids. With an excess ectopic fat accumulation in obesity, metabolic syndrome or diabetes mellitus, however, the deleterious actions of EAT predominate (Konwerski et al. 2022). EAT is strongly associated with atrial fibrillation (AF) burden and outcome after ablation, and after adjustment for left atrial volume and body mass index, periatrial EAT depots of patients with AF exhibited significantly higher levels of CD54 (Girerd et al. 2013), also known as intercellular adhesion molecule (ICAM)-1. Spatio-temporal resolution of adhesion molecules in obese mouse WAT identified CD54/ICAM-1 as being particularly critical for pro-inflammatory cellular dynamics linked with leukocyte activation (Nishimura et al. 2008). CD54 is elevated in the circulation and visceral fat of obese mice and humans (Brake et al. 2006, Bošanská et al. 2010) and is causally linked with manifestation of diabetes (Molina-Ayala et al. 2022). Our finding that EAT from obese patients was more abundant in PAR4 and both CD68 and CD54 suggests that the causal interaction between PAR4 and WAT inflammation seen in mice also occurs in the clinical context of metabolic syndrome.

**Fig. 8:**
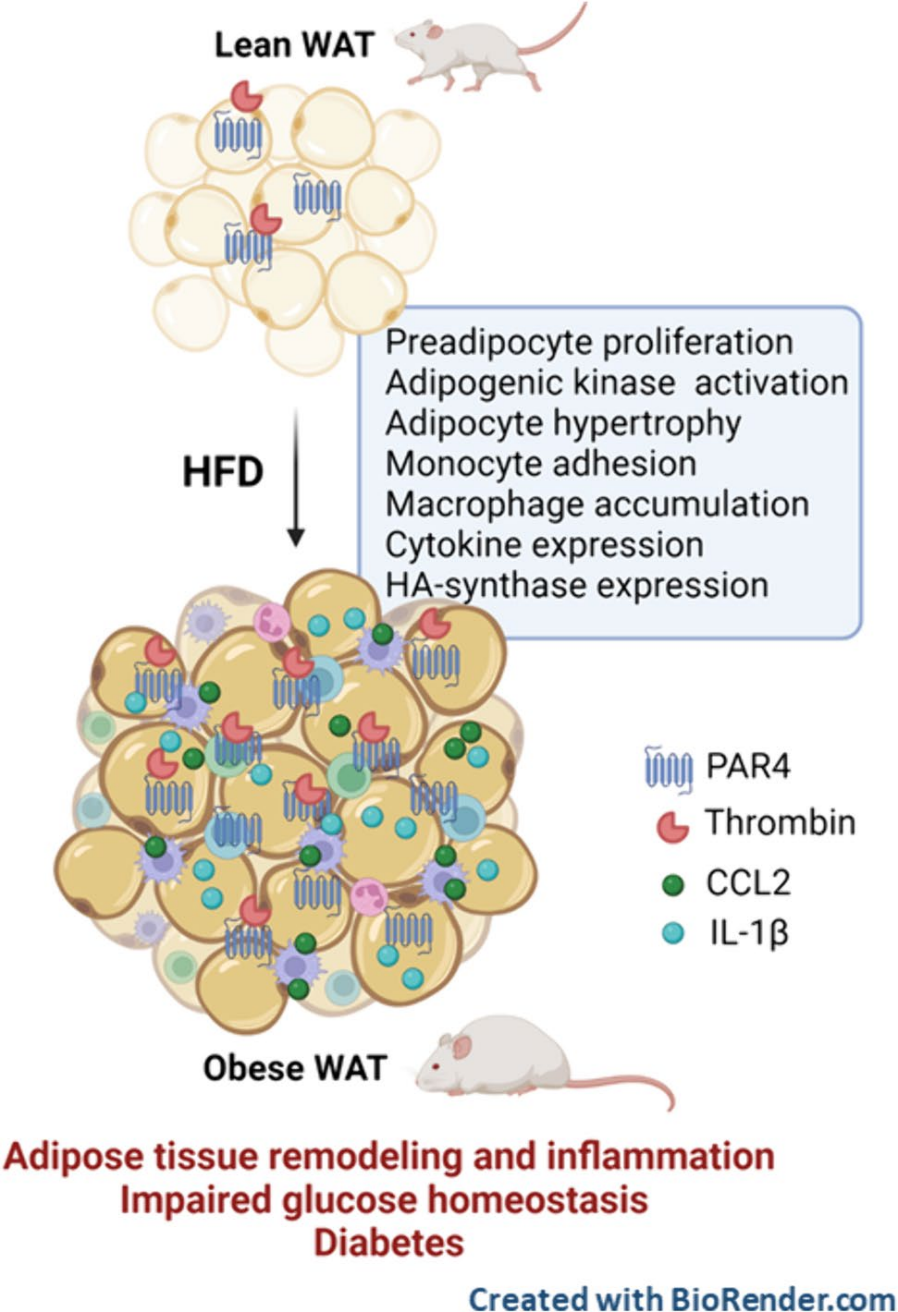
Schematic summary of findings**.** In the lean state, low-level PAR4 expression is constitutively present in visceral white adipose tissue (WAT), preadipocytes and adipocytes. With developing obesity and the associated hypercoagulant state, increased local thrombin together with progressively upregulated PAR4 expression promote preadipocyte hyperplasia, adipogenic kinase activation and monocyte attachment. In expanding obese WAT, abundant PAR4 expression and activation contribute to adipocyte hypertrophy and sustained inflammation typified by macrophage accumulation, adipocytokine expression and upregulation of hyaluronan (HA) synthase. In the absence of PAR4, WAT expansion and inflammation are blunted, and glucose tolerance is improved, and in consequence, the transition to diabetes will be delayed

In conclusion, we have identified PAR4 as a driver of thrombo-inflammation and adipose tissue expansion during obesity development. A schematic summary of the main findings is provided in Fig. 8. PAR4 activation by locally formed thrombin may facilitate a sustained preadipocyte proliferation, hypertrophy of maturing adipocytes and the creation of a pro-inflammatory milieu that contributes to loss of metabolic homeostasis. The distinct nature of PAR4-signalling in human versus mouse platelets (Renna et al. 2023) is a major translational hurdle to consider in the context of our study, although species-specific differences relating to adipocyte PAR4 function have not been reported. Novel small-molecule PAR4 antagonists (BMS-986120, BMS-986141) have been evaluated in phase I randomised, double-blind, placebo-controlled single- and ascending-dose clinical studies, and shown to be well-tolerated, with dose-proportional pharmacokinetics and pharmacodynamics in healthy participants over a wide dose range (Merali et al. 2022, Merali et al. 2023). Our findings warrant further validation to ascertain if these emerging PAR4 antagonists can provide additional therapeutic benefits, beyond their canonical antiplatelet action, in the context of adipose tissue inflammation and remodeling.

## Data Availability

Data and materials may be available from the corresponding author at reasonable request.
